# Impact of Latent Infection Treatment in Indigenous Populations

**DOI:** 10.1371/journal.pone.0071201

**Published:** 2013-07-31

**Authors:** Lucia Suemi Yuhara, Flávia Patussi Correia Sacchi, Julio Croda

**Affiliations:** 1 Sergio Arouca National School of Public Health, Oswaldo Cruz Foundation, Rio de Janeiro, Rio de Janeiro, Brazil; 2 Department of Health of Coronel Sapucaia, Mato Grosso do Sul, Brazil; 3 University Hospital, Federal University of Grande Dourados, Brazil; 4 Faculty of Health Sciences, Federal University of Grande Dourados, Brazil; McGill University, Canada

## Abstract

The aims of the present study were to identify risk factors associated with latent tuberculosis (TB), examine the development of active disease among contacts, and assess the effectiveness of treating latent infection in indigenous Brazilians from January 2006 to December 2011. This was a retrospective study consisting of 1,371 tuberculosis contacts, 392 of whom underwent treatment for latent infection. Morbidity-from-TB data were obtained from the Information System for Disease Notification (SINAN) database, and the contacts’ data were collected from the clinical records using forms employed by Special Department of Indigenous Health (SESAI) multidisciplinary teams, according to SESAI’s instructions. The variables that were associated with latent infection among the contacts were age (odds ratio [OR]: 1.03; 95% confidence interval [CI]: 1.02–1.04) and close contact with a smear-positive index case (OR: 2.26, 95% CI: 1.59–3.22). The variables associated with the development of active TB among the contacts were a tuberculin skin test (TST) ≥10 mm (relative risk [RR]: 1.12, 95% CI: 1.07–1.17), age (RR: 1.01, 95% CI: 1.00–1.03), and treatment of latent infection (RR: 0.03, 95% CI: 0.01–0.27). The estimated number of latent infection treatments needed to prevent one case of active TB among the contacts was 51 treatments (95% CI: 33–182). In contacts with TST ≥10 mm, 10 (95% CI: 6–19) latent infection treatments were necessary to prevent one case of active TB. Age and close contact with a smear-positive index case were associated with latent TB. Screening with TST is a high priority among individuals contacting smear-positive index cases. Age and TST are associated with the development of active TB among contacts, and treatment of latent infection is an effective measure to control TB in indigenous communities.

## Introduction

Over the past 500 years, Brazil’s indigenous population has been affected by a tuberculosis (TB) epidemic characterized by frequent treatment failure and high mortality rates [1], [2], [3], [4], [5], [6], [7], [8]. Social and environmental determinants such as illiteracy, low income, high malnutrition index, intestinal worms, alcoholism, poorly ventilated and illuminated dwellings, and a high density of people per household are closely associated with the high transmissibility and incidence of TB in these communities [1], [2], [3], [4], [5], [6], [7], [8], [9], [10], [11], [12], [13], [14], [15]. In Dourados, work at sugarcane mills, a history of contact with individuals with TB, illiteracy, male sex, and lack of home ownership were identified as risk factors for TB [10]. Despite the implementation of directly observed therapy (DOTS) and a significant decrease in incidence from 700 to 260 cases per 100,000 inhabitants in the indigenous populations of Dourados [9], new interventions are needed to control the disease.

The source of TB infection is often an individual with the pulmonary form of the disease. One single source is estimated to infect an average of 10 to 15 contacts within the community per year [16]. Greater frequency and longer duration of contact with bacilliferous cases are associated with higher proportions of infected individuals [17]. The risk of progression from infection to disease is approximately 10%, and approximately half of this risk occurs within the first two years of infection [18], [19]. The identification and treatment of latent tuberculosis infection provides preventative care to the infected individual and improves the collective public health [20], [21]. Regimens of isoniazid monotherapy have been shown to prevent TB in diverse categories of patients [22], [23], [24]. However, self-supervised daily isoniazid regimens have completion rates of 60% or less in typical settings, attributable largely to the regimen’s duration of ≥6 months [20], [25].

In the indigenous population, treatment of latent tuberculosis infections is recommended if an individual presents a tuberculin skin test ≥5 mm independent of age and vaccination status after active tuberculosis disease is ruled out [18]. It is crucial to evaluate the impact of latent infection treatment in vulnerable populations with a high disease rate when planning future interventions for TB control. This retrospective study is the first to identify risk factors associated with latent TB and the development of active TB in indigenous contacts.

## Methods

### Study Site and Design

The present study was conducted at Amambai and Dourados, the two largest health centers in the State of Mato Grosso do Sul (Brazil). These health centers are responsible for providing health care for 11 villages distributed across six municipalities; this corresponds to a total indigenous population of 25,208 individuals, which represents 58.8% of the Guarani-Kaiowa people and 36.6% of the indigenous population of that state. A retrospective cohort study was conducted using data collected from patients with TB and their contacts from January 2006 to December 2011.

The data analysis periods in the two health centers differed and are consistent with the Special Department of Indigenous Health’s (Secretaria Especial de Saúde Indígena - SESAI) initiation records at these two centers. At Amambai Health Center, data were collected between 2006 and 2010; at Dourados Health Center, data were collected between 2009 and 2011.The SESAI is responsible for overall community health management and for administering the tuberculosis control program for the indigenous population. Local health centers provide technical and administrative support to the Multidisciplinary Teams of Indigenous Health.

### Exclusion and Inclusion Criteria

All of the indigenous cases of tuberculosis were entered into the Information System for Disease Notification (Sistema de Informação de Agravos de Notificação - SINAN), and the data for the contacts that underwent tuberculin skin tests at the Health Centers of Amambai and Dourados from January 2006 to December 2011 were included. The diagnosis of pulmonary TB was based on clinical and epidemiological data, X-ray, thoracic computed tomography (CT), and/or positive sputum smear or culture. The diagnosis of extrapulmonary TB was based on clinical and epidemiological data and complementary tests, such as cerebrospinal fluid (CSF) analysis, CT, and/or biopsy. The cultures were performed in solid medium, and detection and susceptibility testing were performed using the Mycobacterial Detection System-BACTEC™ MGIT™ 960 (BD: Becton, Dickinson and Company, Franklin Lakes, NJ, USA). Index cases that were discharged because of a change in diagnosis were excluded.

A contact was defined as any person living in the same environment as an index case at the time of TB diagnosis. Contact interactions took place at home, at work, in long-term stay institutions, at school, or at preschool. Evaluations of the degree of contact exposure considered the individualized form of the disease, the environment, and the exposure time. When a single patient or contact was identified twice, the less-complete record was excluded. We also excluded contacts without TST values, without a case name index, and who did not live in Dourados or Amambai.

### Data Collection

Morbidity data were obtained from the SINAN database of the state Tuberculosis Control Program/State Department of Health (PCT/SES) database. Population data were collected from the National Health Foundation (Fundação Nacional da Saúde - FUNASA) website. The contacts’ data were collected from the clinical records using the forms employed by the SESAI-PCT Multidisciplinary Teams of Indigenous Health according to the organization’s instructions. For TB cases, retrospective data collection was limited to information available in routine records: clinical form, date of diagnosis, smear results, date of the beginning and end of treatment, and HIV status. For contacts: sex, age, BCG scar, TST value, symptoms of TB, previous TB, X-ray, sputum smear and culture results, name of index case, data for latent infection treatment, and number and dates of missed doses.

### TST Testing and Treatment of Latent Infection

TST was performed in a two-step protocol [20] by a trained nurse from SESAI; all indigenous contacts with initial negative TST underwent repeat TST testing at 7–14 days to determine the need for boosting because of the anticipated need for repeat testing. Two tuberculin units (0.1 ml) of RT23 PPD (Staten Serum Institut, Copenhagen) were injected intradermally into the volar aspect of the left forearm. After 48–72 hours, the maximum diameter of palpable induration was measured by a trained TST reader. The TST was considered positive if the induration was ≥10 mm (after the two-step protocol). All adverse events were recorded.

In the indigenous reserve, DOT is supervised by indigenous health agents who provide the drug treatment 5 days per week for 6 months. Leaders in the community are identified and trained to be health agents [26]. Any close-contact asymptomatic individual with a tuberculin skin test ≥5 mm was a candidate for treatment. Newborn cohabitants of index cases were not vaccinated with BCG at birth. Isoniazid was administered for 3 months, and after this period, the TST was performed. If the result was ≥5 mm, chemoprophylaxis was continued for another 3 months; otherwise, isoniazid use was interrupted and the patient was vaccinated with BCG. Treatment was considered complete when at least 80% of the recommended doses of isoniazid were administered over a period of 6–9 months.

### Data Analysis

The analysis was conducted with the aim of identifying risk factors associated with latent TB and the development of active TB in indigenous contacts. The contacts were stratified in a cross-sectional design based on the TST results to estimate the ORs associated with latent TB. In a second phase, the contacts were stratified based on the completeness of latent TB treatment in a retrospective cohort design. The data corresponding to the assessed variables were entered into the Epi-Data software database, Version 3.0, and analyzed using the statistical software SAS Version 9.1 (SAS Institute Inc., Cary, NC, USA).

The incidence of TB in contacts was calculated as the number of TB cases among contacts stratified according to the tuberculin skin test results (0–4 mm, 5–9 mm, and ≥10 mm) divided by the total number of contacts in each category. The interval between the diagnosis of the index case and the diagnosis of active TB in the contacts was calculated based on the dates recorded in the SINAN notification form by subtracting the contact’s TB diagnosis date from the index case’s date of diagnosis. The contacts were then divided into the following diagnosis interval categories: less than 30 days, 30–180 days, and more than 180 days.

To calculate the number of latent infection treatments necessary to prevent one case of active tuberculosis and the same number adjusted for age and tuberculin skin test results, the following equation was used: NNT  = 1/A – B, where NNT = number of necessary treatments, A = proportion of contacts without latent infection treatment who developed TB disease, and B = proportion of contacts with latent infection treatment who developed TB disease.

Each variable was initially evaluated to ascertain its distribution pattern, which was used to select the statistical procedure to apply. Variables with nonnormal distributions were assessed using non-parametric tests, and variables with normal distributions were evaluated using parametric tests. Dichotomous and categorical data were analyzed using the chi-squared test or Fisher’s exact test when the number of subjects per cell was less than five.

Bivariate analysis was performed to investigate the association between the independent and dependent variables. Variables that were statistically significant (p<0.20) were included in the multivariate analyses used to assess the joint effect of the potential risk factors associated with latent TB and the development of active TB in indigenous contacts.

Regarding the variables associated with latent tuberculosis, we used a logistic regression analysis to estimate the crude and adjusted ORs for tuberculin exposure and treatment outcomes in a cross-sectional approach. The variables for the final model were selected in a stepwise manner, in which the best regression equation was selected using backward elimination. The independent variables that maintained an association with latent TB after adjustment (p≤0.05) were retained in the model.

For the variables associated with the development of active TB, we used a Poisson regression model to estimate the crude and adjusted relative risk (RR) of the various variables [27].

### Ethical Considerations

This study was approved by the Human Research Ethics Committee of the Sergio Arouca National School of Public Health (226/1) and the National Commission for Ethics in Research (048/2012) with a waiver of informed consent, as this was a retrospective study based on data supplied by SINAN, the municipalities’ PCT, the state’s PCT, the SESAI’s PCTs, and the health centers of Amambai and Dourados. Authorization to use the data from the registry of TB cases was requested from the chair of the Indigenous Special Sanitary District (Distrito Sanitário Especial Indígena, DSEI) of Mato Grosso do Sul, as this institution is responsible for the disease records of affected members of indigenous populations, with support from the Indigenous Health Teams of SESAI and SES. Additionally, the General Coordination of Studies and Research of the National Indian Foundation (Fundação Nacional do Índio, FUNAI) (Process 2388–12) granted authorization to enter the indigenous reservation to review the clinical records in the indigenous health centers. This study was approved by the Indigenous District Council (Conselho Distrital Indígena, CONDISI), which is composed of the leaders of the indigenous peoples in the state.

## Results

Between January 2006 and December 2011, 1,039 TB cases were reported to SINAN. In total, 527 cases were excluded; 13 of these cases did not have a confirmed diagnosis of TB, and 514 were nonindigenous people, according to the respondents’ self-reports. In total, 512 TB cases were reported in Dourados, with an annual diagnosis rate of 340/100,000 inhabitants. Among the index TB patients, most (62.5%) were aged 20 to 49 years, were male (66.8%), and had no formal schooling (94.0%). Most of the cases exhibited pulmonary TB (90.2%). A sputum smear was performed in 85% of the cases, and 59.9% were positive for acid-fast bacilli. Sputum cultures were performed in 62.9% of the cases; positive results were obtained in 79.5% of the cultures. Additionally, 3.0% of the cases tested positive for human immunodeficiency virus (HIV), and the most frequent TB treatment outcome (90.3%) was cure and discharge.

**Figure 1:**
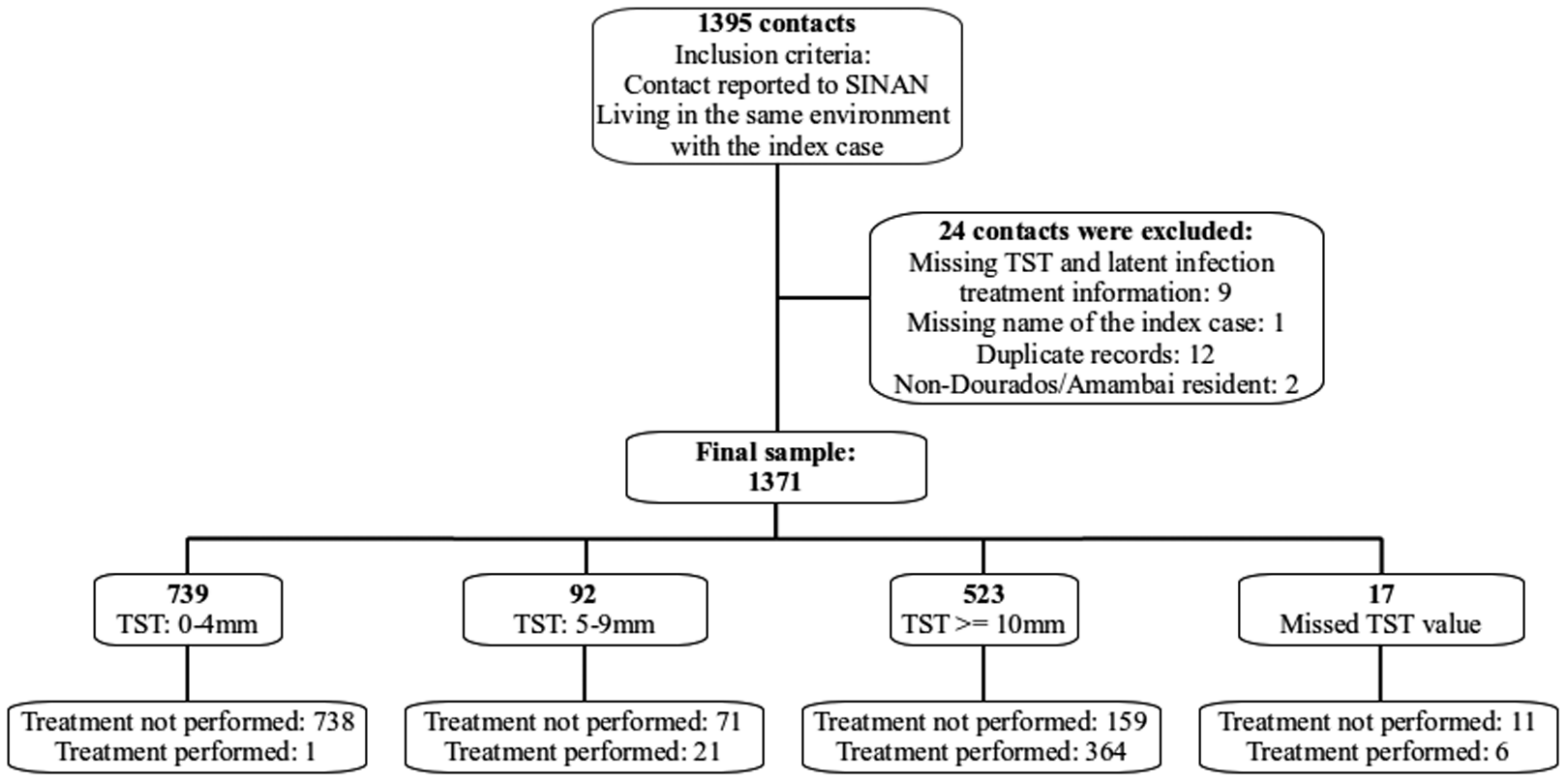
Flowchart illustrating the recruitment of tuberculosis contacts.

Between January 2006 and December 2011, 1,395 contacts were reported to SINAN. In total, 24 contacts were excluded; among these contacts, nine records did not have TST values, one did not include the name of the index case, 12 were duplicate records, and two were for patients who did not live in Dourados or Amambai. In total, 1,371 contacts were examined; 831 had negative TSTs, 523 had TST ≥10 mm, and 392 (29%) received treatment for latent infection based on SESAI records (Figure 1). During LTBI treatment, two individuals did not complete the treatment, one died of causes unrelated to tuberculosis, and one showed intolerance to isoniazid. Most of the contacts were female (56.2%) and aged 5 to 19 years (47.6%), and the mean length of follow-up was 15.1±35.9 months. Some of the characteristics of the infected and noninfected contacts were similar: approximately 60% were female, more than 90% had BCG scars, and less than 5% were associated with HIV-positive index cases. The mean age of the infected contacts (22.5 years) was higher than that of the noninfected contacts (14.5 years old). The index case was smear-positive in 82% of the infected contacts and 68% of the noninfected contacts (Table 1). According to logistic regression, the contacts’ risk of developing a latent infection increased 3% for each additional year of life. In addition, when the index case was smear-positive, the contacts’ odds of exhibiting latent infection were 2.26 times higher (Table 1).

**Table 1:** Demographic and clinical factors associated with latent tuberculosis among indigenous individuals in contact with tuberculosis cases in Brazil in 2006–2011 (N  = 1,354).

Contact characteristics	Noninfected contacts(n = 831)^a^ *n/N (%)*	Contacts with TBLI(n = 523)^b^ *n/N (%)*	P-value	Crude OR (95% CI)	Adjusted OR (95% CI)
Sex					
Male	380/831 (45.7%)	211/523 (40.3)	0.052^c^	0.80 (0.64–1.00)	
Female	451/831 (54.3%)	312/523 (59.7%)			
BCG					
Yes	753/780 (96.5%)	323/342 (94.4%)	0.103^c^	0.61 (0.33–1.11)	
No	27/780 (3.5%)	19/342 (5.6%)			
Age, mean ± standard deviation	14.5±15.7	22.5±18.5	<0.001^d^	1.03 (1.02–1.04)	1.03 (1.02–1.04)
Smear-positive index case					
Yes	503/735 (68.4%)	361/439 (82.2%)	<0.001^c^	2.14 (1.60–2.85)	2.26 (1.59–3.22)
No	232/735 (31.6%)	78/439 (17.8%)		1.0	
Pulmonary TB index case					
Yes	747/788 (94.8%)	440/460 (95.7%)	0.499^c^	1.21 (0.70–2.09)	
No	41/788 (5.2%)	20/460 (4.3%)		1.0	
HIV-positive index case					
Yes	16/481 (3.3%)	12/303 (4.0%)	0.641^c^	1.20 (0.60–2.60)	
No	465/481 (96.7%)	291/303 (96.0%)		1.0	

Abbreviations: CI, confidence interval; OR, odds ratio; LTBI, latent tuberculosis infection; HIV, human immunodeficiency virus; BCG, Bacillus Calmette-Guérin.

a Data corresponding to 2006–2010.

b Data corresponding to 2009–2011.

c Chi-squared test.

d Student’s t-test.

Contacts with TST ≥10 mm exhibited a 3.96-fold higher probability of developing TB disease than contacts with TST ≤4 mm. Contacts with TST of 5–9 mm exhibited a 4.82-fold higher probability of developing TB disease than contacts with TST ≤4 mm (Table 2). There was no statistically significant difference between contacts with TST of 5–9 mm and those with TST ≥10 mm (RR 1.22, CI 0∶36 to 4∶16).

**Table 2:** Incidence of tuberculosis among 1,371 indigenous contacts according to tuberculin skin test (TST) results.

Tuberculin skin test results	Diagnosis of tuberculosis (n = 23) *n/N(%)*	RR (95% CI)
Not performed	1/17 (5.90)	–
0–4 mm	5/739 (0.68)	Reference
5–9 mm	3/92 (3.26)	4.82 (1.17–19.83)
≥10 mm	14/523 (2.68)	3.96 (1.43–10.92)

Abbreviations: RR, relative risk.

In the present study, TST ≥10 mm, age, and untreated latent infection were associated with the development of TB disease in tested contacts. The risk of TB disease increased by 12% with each additional millimeter of TST and by 1% with each additional year of life, whereas the treatment of latent infection reduced the odds that the contacts would develop TB by 97%. Regarding the interval between the index case’s diagnosis and the contact’s development of active disease, 13.0% of contacts were diagnosed with active TB in less than 30 days, 34.8% were diagnosed between 30 and 180 days, and 52.2% were diagnosed more than 180 days later (Table 3).

**Table 3:** Demographic and clinical factors associated with the development of tuberculosis among the indigenous individuals in contact with tuberculosis patients in Brazil in 2006–2011 (N  = 1,371).

Characteristics	Nontuberculosis(n = 1,348) n/N (%)	Tuberculosis(n = 23) n/N (%)	P-value	Crude RR (95% CI)	Adjusted RR (95% CI)
TST-positive (≥10 mm)	509/1,332 (37.6%)	14/22 (63.6)	0.015^a^	1.06 (1.02–1.111)	1.12 (1.07–1.17)
Male sex	591/1,348 (43.8%)	9/23 (39.1%)	0.651^a^		
BCG	1,069/1,089 (98.2%)	46/46 (100%)	0.354^a^		
Age, mean ± standard deviation	17.5±17.2	28.0±24.5	0.004^b^	1.02 (1.01–1.04)	1.01 (0.99–1.03)
Smear-positive case index					
Yes	863/1,169 (73.8%)	16/21(76.2%)	0.807^a^	1.13 (0.42–3.91)	
No	306/1,155 (26.2%)	5/21 (23.8%)			
HIV-positive index case					
Yes	27/780 (3.5%)	1/17 (5.9%)	0.592 c		
No	753/780 (96.5%)	16/17 (94.1%)			
Treatment of LTBI					
Yes	392/1,417 (28.0%)	1/23 (4.3%)	0.012 c	0.12(0.02–0.88)	0.03 (0.01–0.27)
No	1,003/1,417 (72.0%)	22/23 (95.7%)		1	1
Interval between index case diagnosis and onset of contact treatment					
<30 days		3/23 (13.0%)			
30–180 days		8/23 (34.8%)			
>180 days		12/23 (52.2%)			

Abbreviations: CI, confidence interval; RR, relative risk; TST, tuberculin skin test; LTBI, latent tuberculosis infection; HIV, human immunodeficiency virus; BCG, Bacillus Calmette-Guérin.

a Chi-squared test.

b Student’s t-test.

c Fisher’s exact test.

The treatment of latent infection in contacts reduced the absolute risk of developing active TB by 1.9%, and 51 treatments were necessary to prevent one case of active disease. For contacts over 50 years of age, 11 people had to be treated to prevent one active case; contacts aged 20–49 years required 30 treatments, and contacts aged 0–4 years required 56 treatments. These findings indicate that the treatment of latent infection is more effective in contacts who are older than 50 years. Regarding TST, the treatment of latent infection was not found to significantly impact contacts with TSTs of 0–9 mm, whereas treatment was effective in contacts with TST ≥10 mm; only 10 treatments were needed to prevent one case of disease in these individuals. Thus, contacts with TST ≥10 mm benefited the most from the treatment intervention (Table 4).

**Table 4:** Crude and adjusted (by age and tuberculin skin test results) estimates of the number of latent infection treatments necessary to prevent one case of tuberculosis.

		Contacts who received LTBI treatment		Contacts who did not receive LTBI treatment			
Estimates	Total	n (%)^a^	n (%) with tuberculosis^b^	n (%)^a^	n (%) with tuberculosis^b^	% reduction of absolute risk (95% CI)	Number of treatments needed to prevent one case (95% CI)
Crude	1,417	392 (28)	1 (0.25)	1003 (72)	22 (2.19)	1.9 (0.5–3.1)	51 (33–182)
Adjusted by age (years)							
0–4	275	51 (18)	0 (0.00)	224 (82)	4 (1.79)	1.8 (0.5–3.5)	56 (28–1,945)
5–19	643	187 (29)	1 (0.53)	456 (71)	6 (1.32)	0.8 (−0.7–2.3)^c^	128 (−143–44)^c^
20–49	373	136 (37)	0 (0.00)	237 (63)	8 (3.38)	3.4 (1.1–5.7)	30 (18–93)
≥50	61	17 (28)	0 (0.00)	44 (72)	4 (9.09)	9.1 (0.6–17.6)	11 (6–168)
Adjusted for TST							
0–9 mm	831	22 (3)	1 (4.55)	809 (97)	7 (0.90)	−3.7 (−12.4–5.0)^c^	−27 (−8–20)^c^
≥10 mm	523	364 (70)	0 (0)	159 (30)	14 (8.80)	8.8 (4.4–13.2)	10 (7–21)

Abbreviations: CI, confidence interval; TST, tuberculin skin test; LTBI, latent tuberculosis infection.

a Proportion of all reported contacts who did not receive LTBI treatment.

b Proportion of contacts with tuberculosis who started treatment versus those who did not start treatment for latent tuberculosis infection.

c When the number of necessary treatments equals 0 (zero), the effect of treatment is nonsignificant.

## Discussion

In this study, the variables associated with latent infection were age (OR: 1.03, 95% CI: 1.02–1.04) and contact with a smear-positive index case (OR: 2.26, 95% CI: 1.59–3.22). Studies conducted in the Philippines on household contacts identified age, exposure to multidrug-resistant TB, cohabitation for longer than 10 years with an index case, and sharing the same room with an index case as risk factors for latent infection [28], [29]. In conclusion, the charge related to the inoculum bacillary load and the intensity of exposure appear to be the major risk factors associated with latent TB infection.

Of the contacts examined in the present study, 1.6% eventually developed active tuberculosis. In two other studies conducted in Brazil, the rate of progression ranged from 2.3% to 3.2% [30], [31]. In countries with a low disease load, the rate of progression ranges from 0.51% to 7.6% [32], [33], [34], [35], [36]. This disparity in the disease progression rate may result from the different lengths of follow-up performed in these studies. In our study, the mean duration of follow up was 15.1±35.9 months, whereas other studies used a duration of 24–60 months [30], [33], [35], [36], [37].

The variables associated with the progression from latent to active TB were TST ≥10 mm (RR: 1.12, 95% CI: 1.07–1.17) and age (RR: 1.01, 95% CI: 1.00–1.03), and the treatment of latent infection had a protective effect (RR: 0.03, 95% CI: 0.01–0.27). Several risk factors associated with the development of TB in contacts were identified, including very young or old age, immunosuppression, malnutrition, no or incomplete treatment of latent infection, household contact, exposure to pulmonary TB with smear-positive and/or culture-positive results, and TST ≥5 mm [29], [38], [39], [40]. Similar to other studies’ findings, we found that TST screening is an effective measure for identifying contacts in indigenous communities who are at high risk for developing active TB.

Only 10 latent infection treatments were needed to prevent one case of active tuberculosis in contacts with TST ≥10 mm, and 97% of the treated contacts were protected. Most of the contacts that developed TB had an onset of symptoms and diagnosis six months after the index case’s diagnosis, which indicates that most cases of disease in contacts may be prevented by treating latent infection. In our study, 52% of the contacts developed TB six months after the index case was diagnosed, and 53% were TST-positive at the time of the investigation (Table 2). The opposite situation was observed in Scotland, where 71.5% of contacts with tuberculosis were diagnosed less than six months after the index case, 19% were diagnosed 6–16 months later, and 9.5% were diagnosed 16 to 24 months later [36]. In indigenous communities, it is necessary to prevent active disease in contacts by promptly treating latent disease.

An important limitation of the present study was the small number of contacts with TST of 5–9 mm (N  = 92), which did not allow us to identify differences in the efficacy of latent infection treatment in contacts with TST of 5–9 mm compared with contacts with TST ≥10 mm. Other limitations include those inherent to retrospective studies, including underreporting, missing data, mistakes in data filling, and variation in the quality of the data recorded in the notification forms.

Although the findings of the present study may not be generalizable to other epidemiological situations, the identification of risk factors associated with latent and active TB in contacts in Dourados and Amambai will likely be relevant to indigenous populations in developing countries who are subject to similar conditions. Brazilian studies have indicated that the impact of TB on indigenous peoples is substantial and has become a public health priority [1], [41].

Previous studies have shown that indigenous or aboriginal people are at a higher risk of TB than nonaborigines [42], [43], [44]. Aborigines have a higher prevalence of predisposing risk factors for TB, such as diabetes, alcohol abuse, and smoking. In addition, socioeconomic factors such as overcrowding and poverty are known to contribute to this burden [45]. In their model, Clark and Vynnycky predicted an increasing contribution of endogenous reactivation to total disease burden over time. The high association between latent infection and an increased risk of disease progression may result in a high TB burden caused by disease reactivation in this community [44]. In British Columbia, decreased rates of TB among aboriginal people have been produced by the expansion of DOT and chemoprophylaxis [42]. Thus, screening for latent TB and prophylactic therapy remain important tools for reducing the risk of progression to TB disease in high-risk communities.

In this context, and based on the individual risk factors of this population, numerous studies have indicated the need to implement prevention and control measures that specifically target the indigenous population [5], [9], [46]. To the best of our knowledge, this study is the first to describe the variables associated with latent infection, risk factors related to active TB in contacts, and the impact of latent TB treatment in an indigenous population. These findings may be relevant for other indigenous communities.
